# Two Opposing Roles of SARS-CoV-2 RBD-Reactive Antibodies in Pre-Pandemic Plasma Samples From Elderly People in ACE2-Mediated Pseudovirus Infection

**DOI:** 10.3389/fimmu.2021.813240

**Published:** 2022-01-11

**Authors:** Kyu-Young Sim, Gwang-Hoon Ko, So-Eun Bae, Kyu Yeong Choi, Jung Sup Lee, Byeong C. Kim, Kun Ho Lee, Mi-Ryoung Song, Sung-Gyoo Park

**Affiliations:** ^1^ College of Pharmacy and Research Institute of Pharmaceutical Science, Seoul National University, Seoul, South Korea; ^2^ School of Life Sciences, Gwangju Institute of Science and Technology (GIST), Gwangju, South Korea; ^3^ National Research Center for Dementia, Chosun University, Gwangju, South Korea; ^4^ BK21-Plus Research Team for Bioactive Control Technology, Chosun University, Gwangju, South Korea; ^5^ Department of Biomedical Science, Chosun University, Gwangju, South Korea; ^6^ Department of Neurology , Chonnam National University Medical School, South Korea; ^7^ Research Team for Bioactive Control Technology, Chosun University, Gwangju, South Korea

**Keywords:** SARS-CoV-2, pre-pandemic samples, cross-reactive antibodies, receptor binding domain (RBD), neutralizing activity

## Abstract

A novel coronavirus designated severe acute respiratory syndrome coronavirus 2 (SARS-CoV-2) emerged and caused an outbreak of unusual viral pneumonia. Several reports have shown that cross-reactive antibodies against SARS-CoV-2 also exist in people unexposed to this virus. However, the neutralizing activity of cross-reactive antibodies is controversial. Here, we subjected plasma samples from SARS-CoV-2-unexposed elderly Korean people (n = 119) to bead-based IgG antibody analysis. SARS-CoV-2 S1 subunit-reactive IgG antibody analysis detected positive signals in some samples (59 of 119, 49.6%). SARS-CoV-2 receptor-binding domain (RBD)-reactive antibody levels were most significantly correlated with human coronavirus-HKU1 S1 subunit-reactive antibody levels. To check the neutralizing activity of plasma samples, the SARS-CoV-2 spike pseudotype neutralizing assay was used. However, the levels of cross-reactive antibodies did not correlate with neutralizing activity. Instead, SARS-CoV-2 pseudovirus infection was neutralized by some RBD-reactive plasma samples (n = 9, neutralization ≥ 25%, P ≤ 0.05), but enhanced by other RBD-reactive plasma samples (n = 4, neutralization ≤ -25%, P ≤ 0.05). Interestingly, the blood plasma groups with enhancing and neutralizing effects had high levels of SARS-CoV-2 RBD-reactive antibodies than the plasma group that had no effect. These results suggest that some SARS-CoV-2 RBD-reactive antibodies from pre-pandemic elderly people exert two opposing functions during SARS-CoV-2 pseudovirus infection. In conclusion, preformed RBD-reactive antibodies may have two opposing functions, namely, protecting against and enhancing viral infection. Analysis of the epitopes of preformed antibodies will be useful to elucidate the underlying mechanism.

## Introduction

Four seasonal human coronaviruses (HCoVs), the alphacoronaviruses HCoV-229E and HCoV-NL63 and the betacoronaviruses HCoV-HKU1 and HCoV-OC43, are globally distributed and usually cause mild upper respiratory tract illness and common cold (1, 2). At the end of 2019, however, a novel coronavirus belonging to the *Betacoronavirus* genus designated severe acute respiratory syndrome coronavirus 2 (SARS-CoV-2) emerged and caused an outbreak of unusual viral pneumonia (2, 3). In addition, the emergence of additional new variants with mutated receptor-binding domains (RBDs) with increased ACE2 binding affinity has produced a health emergency (1, 4, 5). Interestingly, SARS-CoV-2 shares homologous sequences with common coronaviruses. Therefore, there have been various studies to determine whether the immune responses to HCoV infection affect the severity of coronavirus disease 2019 (COVID-19) caused by SARS-CoV-2. A study suggested that HCoV^+^ SARS-CoV-2–infected hospitalized patients had less severe COVID-19 illness with lower odds for intensive care unit admission and higher survival probability than HCoV^–^ SARS-CoV-2–infected hospitalized patients (6). In a study of T cell immunity to SARS-CoV-2, 44% of blood samples of unexposed subjects produced interferon-γ after stimulation by SARS-CoV-2 spike protein, the RBD protein or nucleocapsid protein (7). These studies show that preexisting memory CD4^+^ T cells reactive to HCoVs can cross-react with corresponding homologous sequences of SARS-CoV-2 and can affect COVID-19 patient disease severity (8). In a study of B cell immunity to SARS-CoV-2, it was also identified that there were immunoglobulin G (IgG) antibodies reactive to SARS-CoV-2 spike protein in some unexposed samples (9–11). Furthermore, studies have revealed an increased level of OC43 spike protein-reactive IgG antibodies after SARS-CoV-2 infection, suggesting that preexisting memory B cells targeting the epitope of SARS-CoV-2 homologous with the common cold virus can be boosted by SARS-CoV-2 infection (11, 12). However, there is controversy regarding whether cross-reactive antibodies have neutralizing activity against SARS-CoV-2 (9–12). In previous studies, the cross-reactive IgG antibodies against total spike proteins of SARS-CoV-2 were quantified, and the neutralizing activities of the spike protein cross-reactive IgG antibodies were analyzed (9, 12). Furthermore, homology between HCoV and SARS-CoV-2 is higher in the spike S2 subunit than in the spike S1 subunit. Thus, most of the cross-reactive IgG antibodies target the S2 subunit (9, 10). However, the S1 subunit, especially the RBD, is responsible for the direct binding of the spike protein with ACE2, and antibodies targeting the S1 subunit are the main source of neutralizing activity (13, 14). Thus, the relationship between analyzed cross-reactive antibody levels and neutralizing activities can vary because total spike protein-reactive antibodies have been analyzed (9, 12).

## Materials and Methods

### Plasma Samples

Total plasma samples (n = 119) were collected from Korean elderly people attending Chosun University Hospital and Chonnam National University Medical School in Korea before the COVID-19 pandemic (from June 2014 to June 2019). The donors of the plasma samples do not have any comorbidities. All experiments were performed in accordance with the relevant guidelines and regulations. Information related to their age and sex is reported in **Supplementary Table 1**.

### Cell Lines

HEK293T cells were from the American Type Culture Collection (ATCC, Cat. CRL-3216) and cultured in 10% fetal bovine serum (FBS, HyClone, Cat. SH3008403)-supplemented Dulbecco’s modified Eagle’s medium (DMEM, HyClone, Cat. SH30243.01) at 37°C and with 5% CO_2_. HEK293T-ACE2 cells were established *via* infection of VSV G-pseudotyped lentivirus packaging human ACE2 encoded by pWPI-IRES-Puro-Ak-ACE2 [Addgene, Cat. 154985, kindly provided by Inchan Kwon (Gwangju Institute Science and Technology)]. Pseudotype production was performed as described in the Method Details below. The ACE2 expression level was maintained under 10 μg/ml puromycin. ACE2 expression was confirmed through surface staining (R&D Systems, Cat. FAB933A-100) and inhibition assay by soluble ACE2 protein (*In vivo*gen, Cat. fc-hace2).

### Bead-Based IgG Antibody Analysis

Before bead-based IgG antibody analysis, protein biotinylation was performed with EZ-Link™ NHS-LC-LC-Biotin (*Thermo Fisher* Scientific, Waltham, MA, USA, Cat. 21343) according to the manufacturer’s protocols. Dialysis was conducted using a Slide-A-Lyzer™ MINI Dialysis Device Kit (Thermo Fisher Scientific, Cat. 69558) for removing unreacted biotinylation reagent. Dialysis using PBS was repeated twice at RT for 2 h, followed by dialysis at 4°C for 12 h and dialysis at RT for 1 h with gentle stirring. Biotinylated proteins were stored in 50% glycerol and 0.02% sodium azide buffer at -20°C. HCoV-OC43 spike S1 subunit protein (AcroBIOSYSTEMS, Cat. SIN-V52H5), HCoV-HKU1 spike S1 subunit protein (SinoBiological, Cat. 40021-V08H), HCoV-229E spike S1 subunit protein (SinoBiological, Cat. 40601-V08H), HCoV-NL63 spike S1 subunit protein (SinoBiological, Cat. 40600-V08H), SARS-CoV-2 spike S1 subunit protein (SinoBiological, Cat. 40591-V08H) and SARS-CoV-2 RBD protein (SinoBiological, Cat. 40592-V08B) were used for biotinylation. For bead-based IgG antibody analysis, Dynabeads M-280 Streptavidin (< 200 μg, Invitrogen, Carlsbad, CA, USA, Cat. 11205D) were coated with biotinylated proteins (10 μg/ml) for 2 h at 4°C and washed twice with a solution of 0.1% bovine serum albumin (BSA) (BOVOGEN, *Melbourne, Australia, Cat.* BSAS 0.1) in PBS (pH 7.4) for 5 min at 4°C. After bead isolation, the beads were incubated with plasma samples (diluted 1:400 in PBS/0.1% BSA). After incubation overnight at 4°C, washing was performed twice with PBS (pH 7.4) containing 0.05% Tween-20. Isolated beads were incubated with secondary antibodies (diluted at 0.4 μg/ml in PBS/0.1% BSA), and R-phycoerythrin Affinipure F(ab’)2 fragment goat anti-human IgG antibodies (Jackson ImmunoResearch, *West Grove, PA, USA*, Cat. 109-116-170) were incubated overnight at 4°C. Washing was performed twice with PBS containing 0.05% Tween-20. The beads were analyzed on a BD *FACSCanto II *(*Becton*,* Dickinson and Company*, *Franklin Lakes*, NJ, United States) and analyzed with FlowJo™ v10.7.1 (*Becton, Dickinson and Company*) data analysis software. A serially diluted anti-SARS-CoV-2 RBD neutralizing antibody (AcroBIOSYSTEMS, Cat. SAD-S35) was used to quantify plasma IgG antibodies. Therefore, 1 AU was defined as an amount equivalent to 1 ng of anti-RBD antibody.

### Enzyme-Linked Immunosorbent Assay (ELISA)

Human anti-SARS-CoV-2 S1 RBD IgG antibody ELISAs (BioVendor, Cat. RAI009R) were used to compare the limit of detection (LOD) and limit of quantitation (LOQ) with the bead assay, according to the manufacturer’s protocols. For calculation of the LOD and LOQ, serially diluted anti-SARS-CoV-2 RBD neutralizing antibody (AcroBIOSYSTEMS) was used.

### SARS-CoV-2 Spike Pseudotype Neutralizing Assay and Quantification

Lentiviral SARS-CoV-2 Spike pseudotypes were generated with a spike-pseudotyped lentiviral kit from BEI Resources (Cat. NR-53816) as described previously (15). Briefly, 4.0 × 10^6^ HEK293T cells were cultured overnight and transfected with 10 μg of pHDM SARS-CoV-2-Spike glycoprotein with a C-terminal 21 amino acid deletion (BEI Resources, Cat. NR-53742), a lentiviral backbone with the Luc2 gene (BEI Resources, Cat. NR-52516) and lentiviral packaging plasmids (BEI Resources, Cat. NR-52517, NR-52518, NR-52519) using FuGENE^®^ HD (Promega, Cat. E2312). Thirty-six hours after transfection, the culture medium was refreshed, and the supernatant was harvested 72 h after transfection. The supernatant was clarified by centrifugation at 500×g for 10 min and filtration through a 0.45 μm filter (Advantec, Cat. 25CS045AS). The filtered media were concentrated through a lenti-X™ concentrator (Takara, Cat. 631232) as described in the manufacturer’s guidelines. Lentiviral VSV G pseudotypes packaging human ACE2 were produced under the same platform with a lentiviral backbone plasmid encoding human ACE2 (Addgene, Cat. 154985). These pseudotypes were stored at -80°C until infection.

The titer of lentiviral SARS-CoV-2 pseudotypes that selectively infect HEK293T-ACE2 cells was determined by measuring relative luciferase units (RLUs) as described previously (15). For infection, HEK293T and HEK293T-ACE2 cells were seeded in 50 μl at 1.0 × 10^4^ cells/well in a 96-well cell culture plate. The next day, HEK293T or HEK293T-ACE2 cells were treated with polybrene (5 μg/ml). After the treatment, 1.0 × 10^6^ RLUs/well of pseudoviruses were preincubated with serially diluted plasma, neutralizing antibody or anti-HA antibody, which was a negative control (Sigma-Aldrich, Cat. H9658), for 1 h at 37°C before infection. Forty-eight to sixty hours post infection of the plasma-virus or antibody-virus mixture, a luciferase assay was performed as described in the manufacturer’s protocol (Promega, Cat. E1501).

### Quantification and Statistical Analysis

The significance of differences between two groups was analyzed using unpaired Student’s t-tests. Pearson’s correlation was performed using Origin2021 software (Origin Lab Corporation) to determine associations between two continuous variables. To compare the sensitivity of the bead-based IgG antibody assay with that of the ELISA, the LOD and LOQ were calculated using a serially diluted anti-SARS-CoV-2 RBD neutralizing antibody (AcroBIOSYSTEMS). For the determination of the LOD and LOQ values, the limit of blank (LOB) was considered. The LOB was calculated by the mean of the blank + 1.645 (standard deviation of the blank), and the LOD was calculated by LOD = 3.3 × σ / S, where S is the slope of the calibration curve, and σ is the standard deviation of the Y-intercept. The LOQ was calculated by LOQ = 10 × σ / S (16). Standard dilution series obtained for the ELISA and bead-based IgG antibody analysis were used to determine the LOD and LOQ. For linear regression to obtain a calibration curve, data with a range higher than the LOB were analyzed using Origin2021 software (Origin Lab Corporation). All data are expressed as the mean ± SD. For all statistical tests, *P*-values ≤ 0.05 were considered significant.

## Results

**Figure 1:**
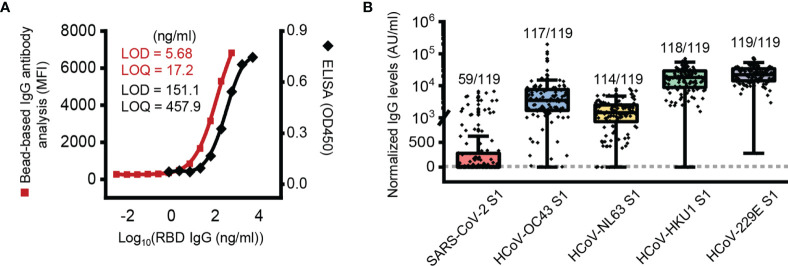
Presence of SARS-CoV-2 S1 subunit reactive antibody in pre-pandemic plasma samples. **(A)** Limit of detection (LOD) and limit of quantification (LOQ) of the ELISA and bead-based IgG antibody analysis. **(B)** Box plot of normalized IgG antibody levels of pre-pandemic samples against each protein. The dotted line represents a threshold set 2-fold above the LOD (11.36 AU/ml). AU, arbitrary unit; 1 AU/ml is equivalent to 1 ng/ml of the anti-RBD antibody.

In this study, we first validated the sensitivity of the bead-based IgG antibody assay. RBD-specific bead-based IgG antibody analysis showed lower LODs (5.68 ng/ml) and LOQs (17.2 ng/ml) than RBD-specific ELISA (LOD = 151.1 ng/ml, LOQ = 456.9 ng/ml) (**Figure 1A**).

With the developed bead-based IgG antibody analysis system, we analyzed plasma samples of Korean elderly people (average age 73.1 ± 5.3, n = 119) collected before the SARS-CoV-2 pandemic outbreak (**Supplementary Table 1**). In order to antibody quantification, RBD-specific IgG antibodies were used as a standard (**Supplementary Figure 1**). In the initial analysis, we used S1 subunit proteins of SARS-CoV-2, HCoV-229E, NL63, HKU1 and OC43 for analysis of S1 subunit-reactive IgG antibodies. In the analysis, we used nonconjugated beads to remove signals from the nonspecific binding of plasma antibodies. The analyzed data showed that the mean signal of HCoV-229E (25,038 AU/ml) was higher than those of HCoV-HKU1 (20,351 AU/ml), HCoV-OC43 (9,574 AU/ml), HCoV-NL63 (1,902 AU/ml), and SARS-CoV-2 (519 AU/ml) (**Figure 1B**). In addition, most elderly people in Korea (more than 95%) have IgG antibodies against the S1 subunit of common coronaviruses, including HCoV-229E (119 of 119; 100%), HCoV-HKU1 (118 of 119; 99.2%), HCoV-OC43 (117 of 119; 98.3%), and HCoV-NL63 (114 of 119; 95.8%). Interestingly, even though we analyzed IgG antibodies with the S1 subunit, there were many positive signals from SARS-CoV-2 S1 subunit-reactive IgG antibody analysis (59 of 119; 49.6%) (**Figure 1B**).

**Figure 2:**
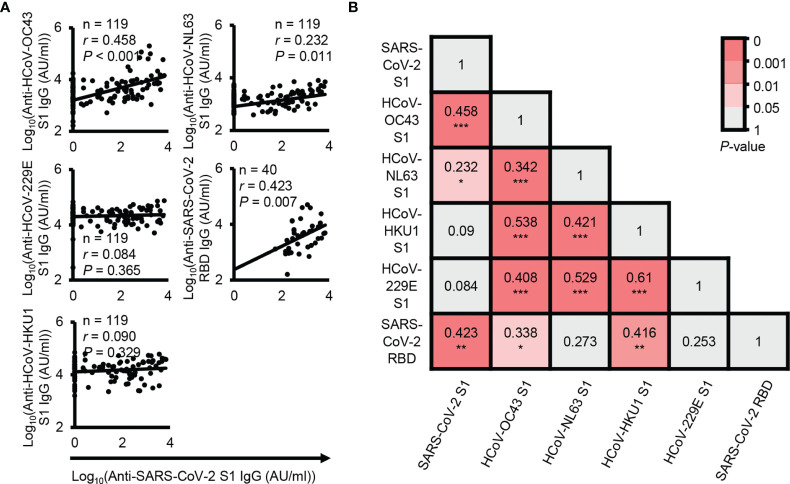
Correlation analysis between SARS-CoV-2 cross-reactive antibody levels and other coronavirus reactive antibody levels. **(A)** Correlation analysis between the levels of plasma IgG antibodies against the SARS-CoV-2 S1 subunit protein and other HCoV S1 subunit proteins. **(B)** Correlation analysis between the levels of plasma IgG antibodies against the SARS-CoV-2 RBD protein and other HCoV S1 subunit proteins. *r* and *P* represent Pearson’s correlation coefficient and its associated *P*-value, respectively. *P ≤ 0.05; **P ≤ 0.01; and ***P ≤ 0.001.

In fact, SARS-CoV-2 shares homologous sequences with common coronaviruses. Thus, to determine which coronaviruses most significantly affect cross-reactive antibodies, we analyzed the correlation between SARS-CoV-2 S1 cross-reactive IgG antibody levels and other coronavirus S1-reactive IgG antibody levels. The results showed that HCoV-OC43 S1 subunit-reactive IgG antibody levels were most highly correlated with SARS-CoV-2 S1 cross-reactive IgG antibody levels (*P* < 0.001, *r* = 0.458) (**Figure 2A**). Thus, our results also showed that HCoV-OC43 infection may mainly contribute to the generation of SARS-CoV-2 cross-reactive antibodies, as shown in a previous study (12). In addition, previous reports have suggested that HCoV-OC43 spike protein-reactive IgG antibodies can affect the disease severity of COVID-19 (11). In addition to S1 subunit-reactive antibody analysis, we also analyzed SARS-CoV-2 RBD cross-reactive IgG antibodies with the plasma samples showing the top 40 SARS-CoV-2 S1 subunit-reactive highest signals for reliable correlation analysis. The levels of SARS-CoV-2 S1 subunit-reactive IgG antibodies had a positive correlation with SARS-CoV-2 RBD-reactive IgG antibody levels (*P* < 0.001, *r* = 0.423) (**Figure 2A**). However, a previous study showed no correlation between SARS-CoV-2 spike protein-reactive IgG antibodies and RBD-reactive IgG antibodies in pre-pandemic plasma samples (12), unlike our data. This discrepancy may be caused by the different analysis systems in which we analyzed SARS-CoV-2 S1 subunit-reactive antibodies. We performed correlation matrix analysis to identify the correlation patterns between HCoV or between HCoV and SARS-CoV-2 S1 subunit-reactive IgG antibodies. Interestingly, the results showed a high correlation between anti-HCoV antibodies (*P* < 0.001, *r* ≥ 0.342), but SARS-CoV-2 S1 subunit-reactive antibodies showed a high correlation only with HCoV-OC43 S1 subunit-reactive IgG antibodies (*P* < 0.001, *r* = 0.423) (**Figure 2B**). In addition, SARS-CoV-2 RBD reactive IgG antibody levels correlated significantly with HCoV-HKU1 S1 subunit reactive IgG antibody levels (*P* < 0.01, *r* = 0.416) (**Figure 2B**). This result possibly indicates that high cross-reactivity between HCoVs can affect protective B cell immunity against different common coronavirus infections. However, antibodies against most HCoV S1 subunits have nonsignificant cross-reactivity with SARS-CoV-2 S1 subunits. For the above reasons, protective B cell immunity against newly emerged SARS-CoV-2 infection may be low in unexposed elderly people.

**Figure 3:**
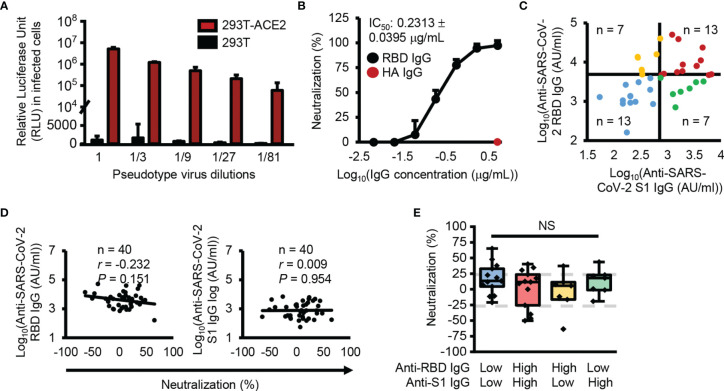
No correlation between antibody levels cross-reactive SARS-CoV-2 S1 subunit or RBD and neutralization activity. **(A)** Infection with SARS-CoV-2 pseudotype viruses. **(B)** Confirmation of RBD specific infection with anti-SARS-CoV-2 RBD (black dots) or anti-HA (red dots) antibodies. **(C)** Dot plot showing the four groups divided using median split (3.63 = log_10_(AU/ml) for RBD and 2.87 = log_10_(AU/ml) for S1 subunit). **(D)** Correlation analysis between anti-SARS-CoV-2 S1 subunit and anti-RBD IgG antibody levels and neutralization. **(E)** Box plot representing the neutralization activity level. Gray dotted lines denote 25% neutralization (upper) and -25% neutralization (lower). Pearson’s correlation coefficient (*r*) and its associated *P*-value (*P*). NS, not significant (unpaired Student’s t-test).

To check the neutralizing activity of SARS-CoV-2 S1 subunit and RBD cross-reactive IgG antibodies, we generated an ACE2-overexpressing HEK293T cell line because ACE2 has been identified as a receptor for SARS-CoV-2 (**Supplementary Figure 2A**). With the generated cell lines, we successfully infected the cells with recombinant pseudotype virus containing SARS-CoV-2 spike proteins, while HEK293T cells were not infected with the recombinant pseudotype virus (**Figure 3A**). In addition, SARS-CoV-2 spike protein-ACE2 specific binding-mediated infection was confirmed by RBD-neutralizing antibody and ACE2 protein-mediated inhibition of the infection (**Figure 3B** and **Supplementary Figure 2B**). In neutralizing activity analysis, we divided the analyzed samples into four groups using median split (3.63 = log_10_(AU/ml) for RBD and 2.87 = log_10_(AU/ml) for S1 subunit) (**Figure 3C**). Interestingly, there were low levels of cross-reactive antibodies against the S1 subunit and high levels of cross-reactive antibodies against the RBD, even though the RBD is a part of the S1 subunit. A previous report also showed a similar result (17, 18), which is possibly caused by hidden epitopes in the S1 subunit structure that are exposed on the RBD without other structural domains of the S1 subunit. Unexpectedly, there was no correlation between neutralizing activity and SARS-CoV-2 S1 subunit and RBD cross-reactive IgG antibody levels (**Figure 3D**) and no difference in neutralizing activity among the four divided groups (**Figure 3E**).

**Figure 4:**
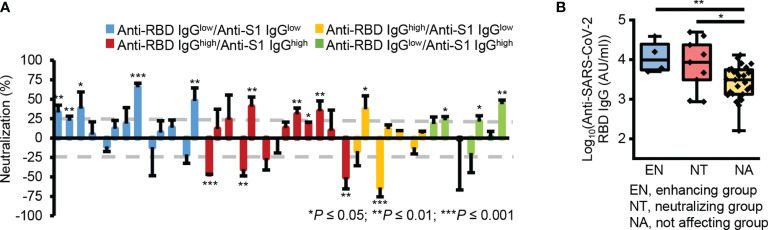
Two opposing roles of RBD-reactive antibodies in SAR-CoV-2 infection. **(A)** Bar graph of the neutralizing activity of individual samples. Gray dotted lines denote 25% neutralization (upper) and -25% neutralization (lower). **(B)** Box plot representing the RBD-reactive antibody levels in three groups according to the nature of ACE2-mediated SARS-CoV-2 viral infection. EN, enhancing group; NT, neutralizing group; NA, not affecting group. *P ≤ 0.05; **P ≤ 0.01; ***P ≤ 0.001 (unpaired Student’s t-test); NS, not significant (unpaired Student’s t-test).

Interestingly, some plasma samples showed significant neutralizing activity (n = 9, neutralization ≥ 25%, *P* ≤ 0.05), while others significantly enhanced pseudotype virus infection (n = 4, neutralization ≤ -25%, *P* ≤ 0.05) (**Figure 4A** and **Supplementary Figure 3**). Besides, the groups which affected the viral infection, showed significantly higher reactivity to RBD (**Figure 4B**). These results reveal there was no correlation between the levels of cross-reactive S1 subunit- and RBD-specific IgG antibodies and neutralizing activity. This may occur because RBD-reactive antibodies in pre-pandemic plasma samples have two opposing functions, namely, inhibiting and enhancing viral infection.

## Discussion

It is controversial whether the preformed B cell immunity to common coronavirus infection affects COVID-19 disease severity. Although the presence of SARS-CoV-2 spike protein cross-reactive IgG antibodies in some pre-pandemic samples is consistently observed in many reports (9, 10, 12), the neutralizing activity of these antibodies is controversial (9, 11, 12, 19). A study measured the neutralizing activity of spike protein cross-reactive IgG antibodies by the SARS-CoV-2 pseudotype virus infection system. However, the cells used for infection were HEK293T cells, so the results of the study may not be mediated by neutralizing ACE2-mediated viral entry (9). In the viral entry of SARS-CoV-2, binding between open-state RBD to ACE2 is required at the first step (20–22), so the ACE2-mediated *in vitro* infection system is required to confirm receptor-specific infection by SARS-CoV-2. Thus, we used the ACE2-mediated pseudotype viral infection system.

In this study, approximately half (49.6%) of pre-pandemic elderly people who live in Korea had SARS-CoV-2 S1 subunit cross-reactive plasma IgG antibodies, which were significantly positively correlated with HCoV-OC43 S1 subunit reactive IgG antibodies. Correlation analysis between HCoV S1 subunit-reactive IgG antibodies showed a high correlation. This correlation suggests the sharing of preformed B cell immunity between common coronaviruses, which may explain why a mild state appears during common coronavirus infection. In our data, the correlation analysis between HCoVs and SARS-CoV-2 S1 subunit-reactive IgG antibodies showed that HCoV-OC43 S1 subunit-reactive IgG antibodies are only significantly related to SARS-CoV-2 S1 subunit-reactive IgG antibodies. Interestingly, when the epitope was narrowed to RBD, SARS-CoV-2 RBD reactive IgG antibody levels also correlated significantly with HCoV-HKU1 S1 subunit reactive IgG antibody levels.

In addition to the detection of cross-reactive antibodies, we also assessed antibody-mediated protective immunity by using a pseudotype virus-mediated *in vitro* infection system. Even though we narrowed down the epitopes, such as using the S1 subunit instead of the total spike protein, the SARS-CoV-2 S1 subunit cross-reactive IgG antibody levels were not correlated with neutralizing activity. Furthermore, the SARS-CoV-2 RBD cross-reactive IgG antibody levels were also not correlated with neutralizing activity. Another important point in this report is that some SARS-CoV-2 S1 subunit cross-reactive IgG antibodies seemed to enhance pseudotype virus infection. This phenomenon was shown in a previous report even though this enhancement was not mentioned in the text (12). In our data, many of the reactive samples (n = 9) significantly inhibited pseudotype virus infection, but some samples (n = 4) significantly enhanced the *in vitro* infection. This result possibly means that specific epitopes are important for neutralizing activity. Thus, based on these data, some HCoV-exposed people may be susceptible to SARS-CoV-2 infections without consideration of preformed T cell immunity. This possibly led to variations of neutralizing activity of RBD-reactive pre-pandemic plasma samples.

A limitation of this study is that only samples from elderly people were analyzed. Thus, the characteristics of the cross-reactive antibodies described in this study cannot be generalized to all pre-pandemic cross-reactive antibodies against SARS-CoV-2 pseudovirus infection, such as those in young people. Another is that this study was unable to analyze for possible age-dependent changes in the function of SARS-CoV-2 cross-reactive antibodies. However, it is important to note that elderly people have been probably infected multiple times by common coronaviruses (23). Thus, plasma samples from the elderly may be suitable for analyzing differences in cross-reactivity of antibodies to the common coronavirus and SARS-CoV-2. In addition, the cross-reactive antibodies are possibly involved in antibody-dependent cell-mediated cytotoxicity and complement-dependent toxicity. However, in this paper, we focused on the neutralizing activity of the cross-reactive antibodies. A future study will be required to characterize the other functions of these cross-reactive antibodies to understand better the roles of cross-reactive antibodies in pre-pandemic samples.

In this report, we found that cross-reactive antibodies against the SARS-CoV-2 S1 subunit were present in the plasma of some elderly Korean people. Interestingly, the levels of cross-reactive antibodies against the SARS-CoV-2 S1 subunit were not correlated with neutralizing activity. Furthermore, even the levels of RBD cross-reactive antibodies were not correlated with neutralizing activity. However, RBD-reactive antibody levels were significantly higher in the groups displaying inhibition and enhancement of viral infection than in the non-affecting group. Thus, our data indicate that the preformed RBD-reactive antibodies have two opposing roles in SARS-CoV-2 infection. Analysis of the epitopes of preformed antibodies will be useful to understand the mechanism by which RBD-reactive antibodies enhance or inhibit SARS-CoV-2 infection in future studies.

## Data Availability Statement

The original contributions presented in the study are included in the article/**Supplementary Material**. Further inquiries can be directed to the corresponding author.

## Ethics Statement

The studies involving human participants were reviewed and approved by institutional review board of Seoul National University (IRB No. E2104/002-012). The patients/participants provided their written informed consent to participate in this study.

## Author Contributions

S-GP conceived this study. S-GP and K-YS designed study and the experiments. M-RS interpreted the data. K-YS, G-HK, and S-EB performed experiments and analyzed the data. K-YS and G-HK wrote the manuscripts. KC, JL, BK, and KL collected patient samples and data. All authors contributed to the article and approved the submitted version.

## Funding

This research was supported by the New Faculty Startup Fund from Seoul National University and by the National Research Foundation of Korea (2021R1A2C3011211).

## Conflict of Interest

The authors declare that the research was conducted in the absence of any commercial or financial relationships that could be construed as a potential conflict of interest.

## Publisher’s Note

All claims expressed in this article are solely those of the authors and do not necessarily represent those of their affiliated organizations, or those of the publisher, the editors and the reviewers. Any product that may be evaluated in this article, or claim that may be made by its manufacturer, is not guaranteed or endorsed by the publisher.
