# Supplementary material for: Two Opposing Roles of SARS-CoV-2 RBD-Reactive Antibodies in Pre-Pandemic Plasma Samples From Elderly People in ACE2-Mediated Pseudovirus Infection
Source: Front Immunol. 2022 Jan 11;12:813240. doi: 10.3389/fimmu.2021.813240 (PMC8787138; doi:10.3389/fimmu.2021.813240)
Supplement: Supplementary file 1 [file DataSheet_1.docx]

Supplementary Material

# Supplementary Tables

**Supplementary Table 1: Dose Fractionation Concepts.**

| **Fractionation** | **Ultracentral Tumors** | **Central Tumors** | **Peripheral Tumors** |
| --- | --- | --- | --- |
| 3 x 15 Gy | - | - | 2* |
| 3 x 9 Gy | - | - | 1 |
| 5 x 10 Gy | - | - | 5 |
| 5 x 6 Gy | 1 | - | - |
| 6 x 5 Gy | 1 | - | - |
| 8 x 5 Gy | 1^#^ | - | - |
| 8 x 7.5 Gy | 1 | 2 | 2 |
| 10 x 5 Gy | 3 | - | - |
| 10 x 5.5 Gy | 2^#^ | - | - |
| 10 x 6 Gy | 1 | - | - |
| 12 x 5 Gy | 1^#^ | - | - |

Gy: Gray
* prescribed to the 65% isodose, ^#^ with one case prescribed to the median dose

**Supplementary Table 2: Dose Constraints to Organs-at-Risk.**

| **Volume Constraint** | **Dose Constraint per Single Fraction** | | | | |
| --- | --- | --- | --- | --- | --- |
|  | **3 Fx** | **5 Fx** | **6 Fx** | **8 Fx** | **10 Fx** |
| **Esophagus** |  |  |  |  |  |
| 0.5 cm³ | < 8.4 Gy | < 6.8 Gy | < 6.0 Gy | < 5.0 Gy | < 4.4 Gy |
| **Spinal Cord** |  |  |  |  |  |
| 0.1 cm³ | < 7.2 Gy | < 5.4 Gy | < 4.8 Gy | < 4.0 Gy | < 3.5 Gy |
| **PBT** |  |  |  |  |  |
| 0.5 cm³ | < 10.7 Gy | < 7.0 Gy | < 6.3 Gy | < 5.5 Gy | < 4.8Gy |
| **Heart** |  |  |  |  |  |
| 0.5 cm³ | < 8.7 Gy | < 5.8 Gy | < 5.3Gy | < 7.5 Gy | < 6.6 Gy |
| **Brachial Plexus** |  |  |  |  |  |
| 0.5 cm³ | < 8.7 Gy | < 5.8 Gy | < 5.3 Gy | < 3.5 Gy | < 3.1 Gy |
| **Thoracic Wall** |  |  |  |  |  |
| 30 cm³ | < 10 Gy | < 7.5 Gy | < 6.8 Gy | < 5.8 Gy | < 5.1 Gy |
| **Great Vessels** |  |  |  |  |  |
| 0.5 cm³ | < 14.3 Gy | < 10.6 Gy | < 9.7 Gy | < 8.0 Gy | < 7.0 Gy |

Fx: fraction, Gy: Gray, CTV: clinical target volume, PBT: proximal bronchial tree.

**Supplementary Table 3: Linear multilevel model for planning target volume (PTV) coverage [%].** There were no divergent transitions.

| **Population Level Effect** | **Prior** | **Posterior** | | | |
| --- | --- | --- | --- | --- | --- |
|  |  | **Mean** | **SD** | **95% CI** | $\hat{\boldsymbol{R}}$ |
| Intercept* | N(90%, 10%) | 90.02 | 0.9 | 88.36 91.91 | 1.00 |
| Plan* [Adaptation] | N(0%, 20%) | 6.3 | 0.53 | 5.27 7.36 | 1.00 |
| Localization [ultracentral] |  | 1.01 | 1.04 | -1.05 3.08 | 1.00 |
| PTV Size  [10 cm³] |  | -0.12 | 0.11 | -0.37 0.07 | 1.00 |
| Plan x Localization* |  | -2.0 | 0.57 | -3.12 -0.87 | 1.00 |
| Plan x PTV Size* |  | -0.12 | 0.05 | -0.23 -0.02 | 1.00 |

SD: standard deviation, CI: compatibility interval, N: normal distribution, PTV: planning target volume, *compatibility interval does not contain 0

**Supplementary Table 4: Linear multilevel model for clinical target volume (CTV) coverage [%].** There were no divergent transitions.

| **Population Level Effect** | **Prior** | **Posterior** | | | |
| --- | --- | --- | --- | --- | --- |
|  |  | **Mean** | **SD** | **95% CI** | $\hat{\boldsymbol{R}}$ |
| Intercept* | N(90%, 10%) | 100.06 | 0.37 | 99.39 100.86 | 1.00 |
| Plan [Adaptation] | N(0%, 20%) | 0.35 | 0.18 | -0.01 0.71 | 1.00 |
| Localization [ultracentral] |  | -0.38 | 0.44 | -1.25 0.50 | 1.00 |
| CTV Size  [10 cm³]* |  | -0.28 | 0.07 | -0.43 -0.17 | 1.00 |
| Plan x Localization |  | -0.11 | 0.2 | -0.51 0.29 | 1.00 |
| Plan x  CTV Size* |  | 0.13 | 0.02 | 0.08 0.18 | 1.00 |

SD: standard deviation, CI: compatibility interval, N: normal distribution, CTV: clinical target volume, *compatibility interval does not contain 0

**Supplementary Table 5: Linear multilevel model for gross tumor volume (GTV) coverage [%].** There were no divergent transitions.

| **Population Level Effect** | **Prior** | **Posterior** | | | |
| --- | --- | --- | --- | --- | --- |
|  |  | **Mean** | **SD** | **95% CI** | $\hat{\boldsymbol{R}}$ |
| Intercept* | N(90%, 10%) | 100.10 | 0.23 | 99.65 100.55 | 1.00 |
| Plan [Adaptation] | N(0%, 20%) | -0.02 | 0.10 | -0.22 0.17 | 1.00 |
| Localization [ultracentral] |  | -0.39 | 0.30 | -0.98 0.20 | 1.00 |
| GTV Size  [10 cm³] |  | -0.07 | 0.05 | -0.18 0.02 | 1.00 |
| Plan x Localization |  | 0.08 | 0.11 | -0.14 0.31 | 1.00 |
| Plan x  GTV Size |  | 0.04 | 0.02 | 0.0 0.07 | 1.00 |

SD: standard deviation, CI: compatibility interval, N: normal distribution, GTV: gross tumor volume, *compatibility interval does not contain 0

**Supplementary Table 6: Linear multilevel model for minimum BED_10_ (BED_min_) inside the planning target volume (PTV).** There were no divergent transitions.

| **Population Level Effect** | **Prior** | **Posterior** | | | |
| --- | --- | --- | --- | --- | --- |
|  |  | **Mean** | **SD** | **95% CI** | $\hat{\boldsymbol{R}}$ |
| Intercept* | N(100Gy,50Gy) | 77.86 | 5.42 | 67.48 88.99 | 1.00 |
| Plan* [Adaptation] | N(0Gy,50Gy) | 9.01 | 1.17 | 6.70 11.29 | 1.00 |
| Localization* [ultracentral] |  | -15.22 | 6.98 | -28.97 -1.23 | 1.00 |
| PTV Size* [10 cm³] |  | -1.57 | 0.49 | -2.55 -0.63 | 1.00 |
| Plan x Localization* |  | -5.21 | 1.27 | -7.71 -2.74 | 1.00 |
| Plan x  PTV Size* |  | 0.37 | 0.12 | 0.14 0.61 | 1.00 |

SD: standard deviation, CI: compatibility interval, N: normal distribution, PTV: planning target volume, *compatibility interval does not contain 0

**Supplementary Table 7: Linear multilevel model for minimum BED_10_ (BED_min_) inside the clinical target volume (CTV).** There were no divergent transitions.

| **Population Level Effect** | **Prior** | **Posterior** | | | |
| --- | --- | --- | --- | --- | --- |
|  |  | **Mean** | **SD** | **95% CI** | $\hat{\boldsymbol{R}}$ |
| Intercept* | N(100Gy,50Gy) | 106.59 | 6.66 | 93.73 120.15 | 1.00 |
| Plan* [Adaptation] | N(0Gy,50Gy) | 6.52 | 1.02 | 4.52 8.54 | 1.00 |
| Localization* [ultracentral] |  | -27.28 | 9.35 | -45.91 -8.81 | 1.00 |
| CTV Size*  [10 cm³] |  | -3.37 | 0.59 | -4.52 -2.22 | 1.00 |
| Plan x Localization* |  | -2.70 | 1.14 | -4.94 -0.46 | 1.00 |
| Plan x  CTV Size* |  | 0.55 | 0.14 | 0.29 0.82 | 1.00 |

SD: standard deviation, CI: compatibility interval, N: normal distribution, CTV: clinical target volume, *compatibility interval does not contain 0

**Supplementary Table 8: Linear multilevel model for minimum BED_10_ (BED_min_) inside the gross tumor volume (GTV).** There were no divergent transitions.

| **Population Level Effect** | **Prior** | **Posterior** | | | |
| --- | --- | --- | --- | --- | --- |
|  |  | **Mean** | **SD** | **95% CI** | $\hat{\boldsymbol{R}}$ |
| Intercept* | N(100Gy,50Gy) | 118.39 | 7.15 | 104.37 132.71 | 1.00 |
| Plan [Adaptation]* | N(0Gy,50Gy) | 2.85 | 0.84 | 1.20 4.49 | 1.00 |
| Localization [ultracentral]* |  | -34.35 | 9.86 | -53.73 -14.88 | 1.00 |
| GTV Size  [10 cm³]* |  | -3.91 | 0.92 | -5.73 -2.11 | 1.00 |
| Plan x Localization |  | -1.55 | 0.96 | -3.44 0.34 | 1.00 |
| Plan x  GTV Size* |  | 0.34 | 0.16 | 0.03 0.65 | 1.00 |

SD: standard deviation, CI: compatibility interval, N: normal distribution, GTV: gross tumor volume, *compatibility interval does not contain 0

**Supplementary Table 9: Linear multilevel model for mean BED_10_ (BED_mean_) inside the planning target volume (PTV).** There were no divergent transitions.

| **Population Level Effect** | **Prior** | **Posterior** | | | |
| --- | --- | --- | --- | --- | --- |
|  |  | **Mean** | **SD** | **95% CI** | $\hat{\boldsymbol{R}}$ |
| Intercept* | N(100Gy,50Gy) | 122.79 | 7.31 | 108.27 137.27 | 1.00 |
| Plan* [Adaptation] | N(0Gy,50Gy) | 1.39 | 0.33 | 0.75 2.04 | 1.00 |
| Localization* [ultracentral] |  | -33.57 | 10.29 | -53.70 -12.75 | 1.00 |
| PTV Size* [10 cm³] |  | -0.68 | 0.19 | -1.05 -0.31 | 1.00 |
| Plan x Localization* |  | -0.72 | 0.36 | -1.42 -0.02 | 1.00 |
| Plan x  PTV Size |  | -0.01 | 0.03 | -0.08 0.06 | 1.00 |

SD: standard deviation, CI: compatibility interval, N: normal distribution, PTV: planning target volume, *compatibility interval does not contain 0

**Supplementary Table 10: Linear multilevel model for mean BED_10_ (BED_mean_) inside the clinical target volume (CTV).** There were no divergent transitions.

| **Population Level Effect** | **Prior** | **Posterior** | | | |
| --- | --- | --- | --- | --- | --- |
|  |  | **Mean** | **SD** | **95% CI** | $\hat{\boldsymbol{R}}$ |
| Intercept* | N(100Gy,50Gy) | 130.45 | 7.90 | 114.70 145.72 | 1.00 |
| Plan* [Adaptation] | N(0Gy,50Gy) | 0.81 | 0.33 | 0.17 1.46 | 1.00 |
| Localization* [ultracentral] |  | -39.33 | 11.30 | -61.29 -16.56 | 1.00 |
| CTV Size*  [10 cm³] |  | -0.50 | 0.24 | -0.98 -0.04 | 1.00 |
| Plan x Localization |  | -0.54 | 0.36 | -1.26 0.18 | 1.00 |
| Plan x  CTV Size |  | -0.01 | 0.04 | -0.09 0.08 | 1.00 |

SD: standard deviation, CI: compatibility interval, N: normal distribution, CTV: clinical target volume, *compatibility interval does not contain 0

**Supplementary Table 11: Linear multilevel model for mean BED_10_ (BED_mean_) inside the gross tumor volume (GTV).** There were no divergent transitions.

| **Population Level Effect** | **Prior** | **Posterior** | | | |
| --- | --- | --- | --- | --- | --- |
|  |  | **Mean** | **SD** | **95% CI** | $\hat{\boldsymbol{R}}$ |
| Intercept* | N(100Gy,50Gy) | 133.64 | 8.83 | 116.04 151.01 | 1.00 |
| Plan [Adaptation] | N(0Gy,50Gy) | 0.57 | 0.31 | -0.05 1.18 | 1.00 |
| Localization [ultracentral]* |  | -42.13 | 12.55 | -66.57 -16.67 | 1.00 |
| GTV Size  [10 cm³] |  | 0.03 | 0.39 | -0.74 0.81 | 1.00 |
| Plan x Localization |  | -0.53 | 0.36 | -1.24 0.18 | 1.00 |
| Plan x  GTV Size |  | -0.05 | 0.06 | -0.17 0.07 | 1.00 |

SD: standard deviation, CI: compatibility interval, N: normal distribution, GTV: gross tumor volume, *compatibility interval does not contain 0

**Supplementary Table 12: Linear multilevel model for the amount of planning target volume exceeding the dose maximum [%].** There were no divergent transitions.

| **Population Level Effect** | **Prior** | **Posterior** | | | |
| --- | --- | --- | --- | --- | --- |
|  |  | **Mean** | **SD** | **95% CI** | $\hat{\boldsymbol{R}}$ |
| Intercept* | N(5%, 5%) | 1.31 | 0.48 | 0.37 2.25 | 1.00 |
| Plan* [Adaptation] | N(0%, 10%) | -0.97 | 0.37 | -1.71 -0.24 | 1.00 |
| Localization [ultracentral] |  | -0.32 | 0.56 | -1.43 0.77 | 1.00 |
| PTV Size [10 cm³] |  | 0.01 | 0.05 | -0.09 0.11 | 1.00 |
| Plan x Localization |  | 0.38 | 0.40 | -0.41 1.16 | 1.00 |
| Plan x PTV Size |  | -0.02 | 0.04 | -0.10 0.05 | 1.00 |

SD: standard deviation, CI: compatibility interval, N: normal distribution, PTV: planning target volume, *compatibility interval does not contain 0

**Supplementary Table 13: Violations of organs-at-risk constraints.**

| **Case** | **PTV Location** | **Dose Fractionation** | **Plan** | **Fraction** | **OAR** | **Initial Distance PTV-OAR** |
| --- | --- | --- | --- | --- | --- | --- |
| # 1 | ultracentral | 10 x 5.5 Gy | Predicted | 1 | Esophagus | Overlap |
|  |  |  |  | 6 | PBT | Overlap |
|  |  |  |  | 8 | PBT |  |
|  |  |  |  | 9 | PBT |  |
|  |  |  |  | 10 | PBT |  |
| # 2 | ultracentral | 10 x 6.0 Gy | Predicted | 1 | Heart | Overlap |
|  |  |  |  | 7 | Heart |  |
|  |  |  | Adapted | 1 | Heart |  |
|  |  |  |  | 1 | Esophagus | Overlap |
| # 3 | ultracentral | 10 x 5.0 Gy | Predicted | 7 | PBT | Overlap |
| # 4 | central | 8 x 7.5 Gy | Predicted | 1 | Aorta | Overlap |
|  |  |  |  | 2 | Aorta |  |
|  |  |  |  | 5 | Aorta |  |
|  |  |  |  | 7 | Aorta |  |
|  |  |  |  | 8 | Aorta |  |
| # 5 | peripheral | 5 x 10 Gy | Predicted | 1 | Plexus | 1 – 2 mm |
|  |  |  |  | 2 | Plexus |  |
|  |  |  |  | 4 | Plexus |  |
|  |  |  |  | 5 | Plexus |  |
| # 6 | peripheral | 5 x 10 Gy | Predicted | 3 | Intestine | > 2 cm |

PTV: Planning Target Volume, OAR: Organ-at-Risk

**Supplementary Table 14: Logistic multilevel model for violation of organs at risk constraints.** This model leaves out interaction terms with the “Plan” variable. There was one divergent transition.

| **Population Level Effect** | **Prior** | **Posterior** | | | |
| --- | --- | --- | --- | --- | --- |
|  |  | **Mean (OR)** | **SD** | **95% CI** | $\hat{\boldsymbol{R}}$ |
| Intercept* | N(0, 2.0) | -3.99 | 1.02 | -6.22 -2.19 | 1.00 |
| Plan* [Adaptation] |  | -1.94 (0.14) | 0.61 | -3.23 -0.83 | 1.00 |
| Localization [ultracentral] |  | 0.17 (1.19) | 1.02 | -1.84 2.26 | 1.00 |
| PTV Volume [10 cm³] |  | -0.24 (0.79) | 0.18 | -0.64 0.08 | 1.00 |

SD: standard deviation, CI: compatibility interval, N: normal distribution, PTV: planning target volume, *compatibility interval does not contain 0

**Supplementary Table 15: Linear multilevel model for planning target volume size [cm³].** There were no divergent transitions.

| **Population Level Effect** | **Prior** | **Posterior** | | | |
| --- | --- | --- | --- | --- | --- |
|  |  | **Mean** | **SD** | **95% CI** | $\hat{\boldsymbol{R}}$ |
| Intercept* | N(75cm³, 32.5cm³) | 48.37 | 10.15 | 28.16 68.36 | 1.00 |
| Fraction* [1] | N(0cm³, 25cm³) | 0.42 | 0.16 | 0.11 0.74 | 1.00 |

SD: standard deviation, CI: compatibility interval, N: normal distribution, *compatibility interval does not contain 0

# Supplementary Figures


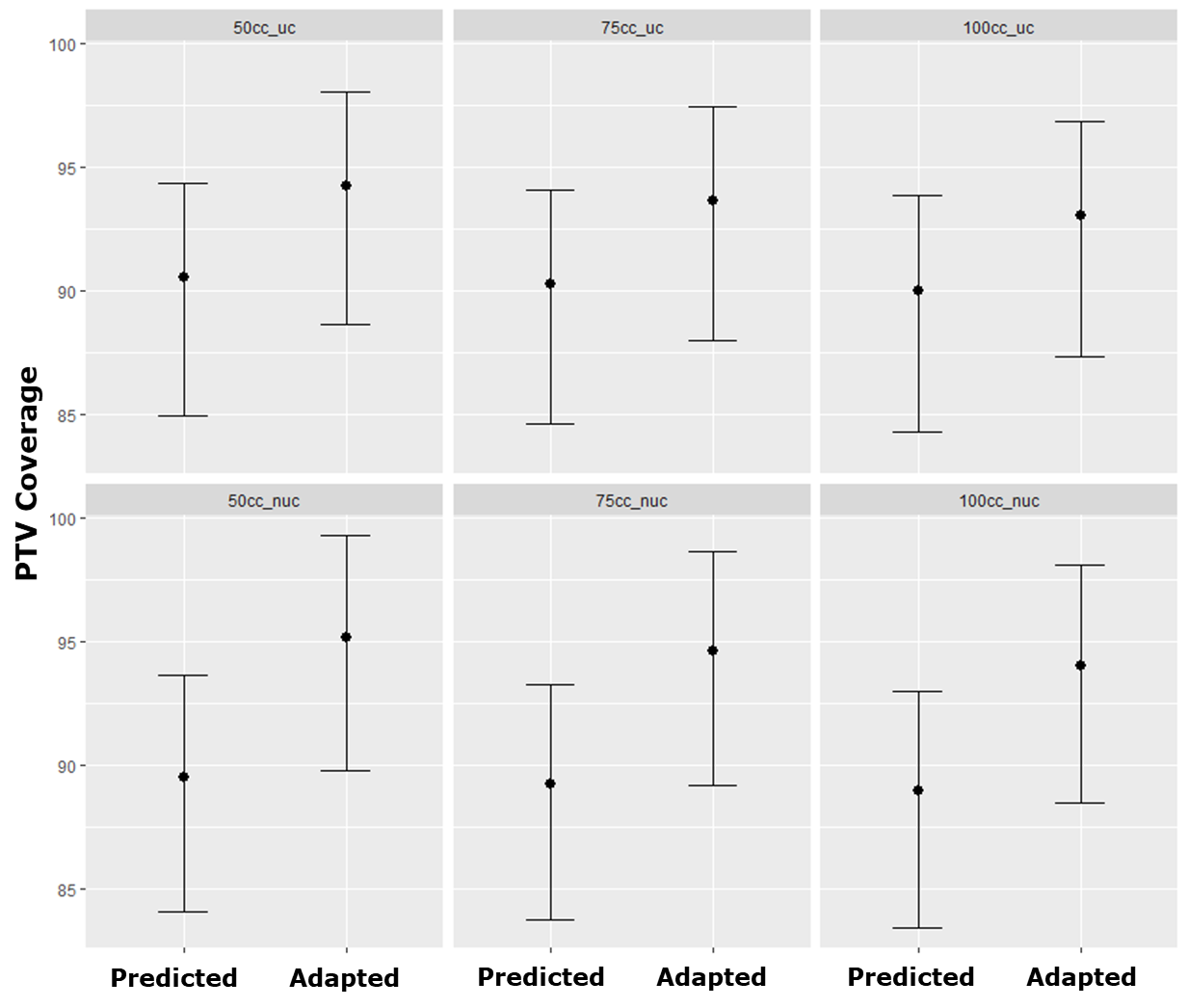


**Supplementary Figure 1: Marginal effects plot for the interaction of planning target volume (PTV) size and ultracentral tumor location with the effect of plan adaptation on the PTV coverage.** Three arbitrary PTV volumes (50 cm³, 75 cm³ and 100 cm³) were chosen. Rows: The positive effect of plan adaptation on PTV coverage is decreased for increasing PTV size. Columns: The positive effect of plan adaptation on PTV coverage is decreased for ultracentral tumor location. (cc: cm³, uc: ultracentral, nuc: non-ultracentral).

**

**

**Supplementary Figure 2: BED_min_ inside the target volumes.** The minimum biologically effective dose (BEDmin, alpha/beta-ratio = 10) is shown for the planning target volume (PTV), clinical target volume (CTV) and gross tumor volume (GTV) before (red) and after (blue) plan adaptation. Fractionation schemes for non-ultracentral tumors allowed a BED_10_ > 100 Gray (Gy), while the most common fractionation given to ultracentral tumors was 10 x 5 -6 Gy which translates to a BED_10_ 75 – 96 Gy.

**

**

**Supplementary Figure 3: BED_mean_ inside the target volumes.** The mean biologically effective dose (BED_mean_, alpha/beta-ratio = 10) is shown for the planning target volume (PTV), clinical target volume (CTV) and gross tumor volume (GTV) before (red) and after (blue) plan adaptation. Fractionation schemes for non-ultracentral tumors allowed a BED_10_ > 100 Gray (Gy), while the most common fractionation given to ultracentral tumors was 10 x 5 -6 Gy which translates to a BED_10_ 75 – 96 Gy.


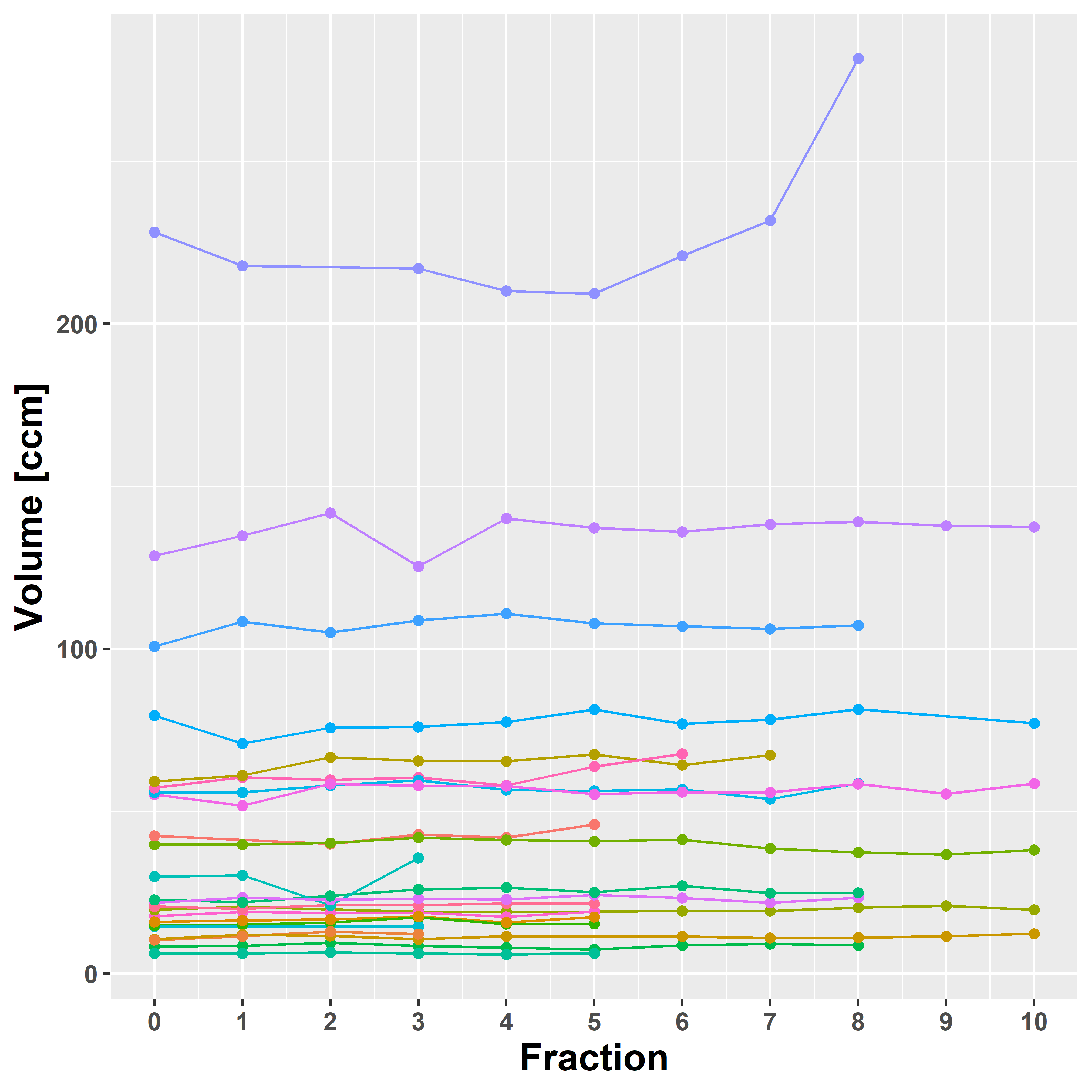


**Supplementary Figure 4:** Development of planning target volume size [cm³] over fractions for each lesion (N = 23).


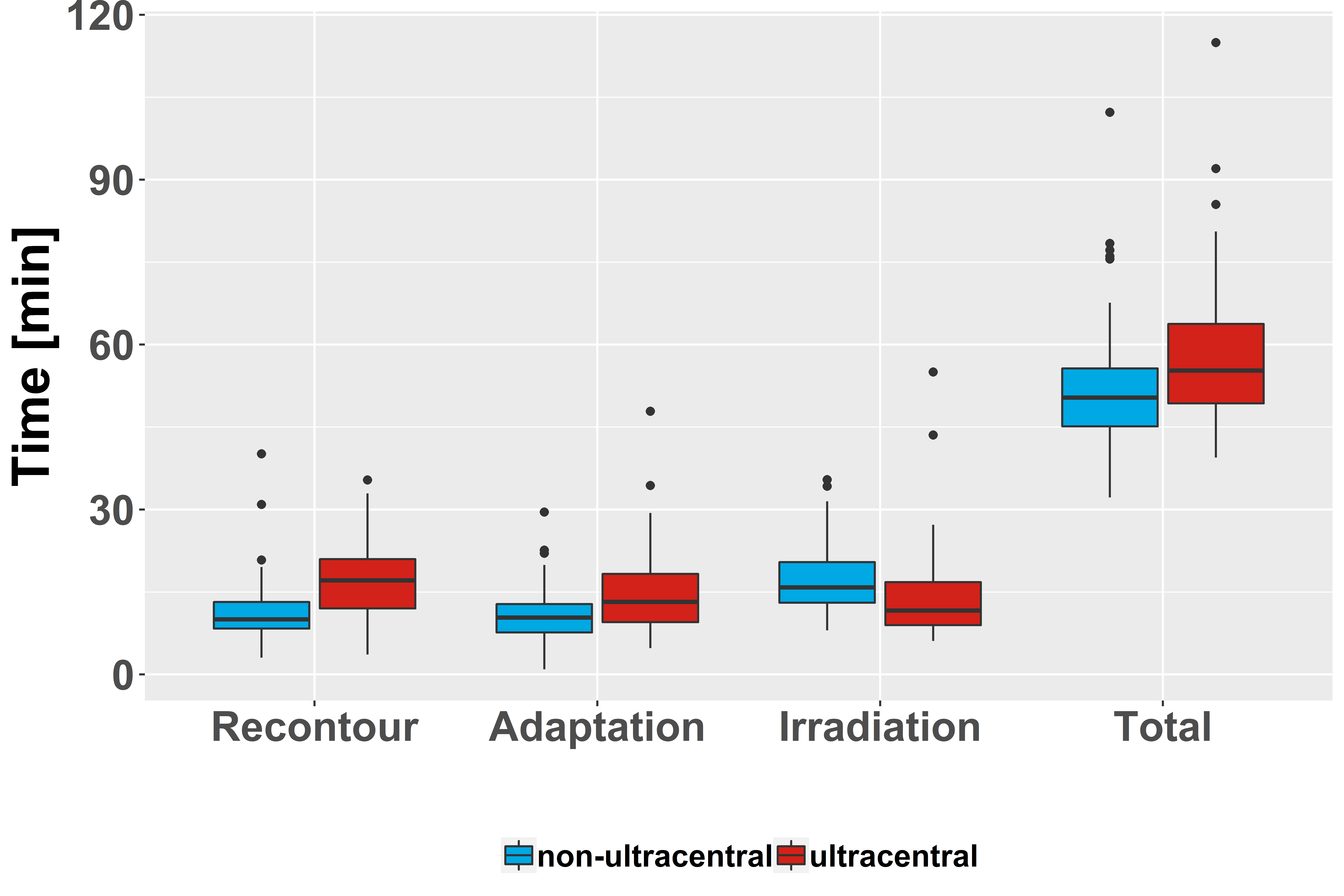


**Supplementary figure 5:** Time requirements for online plan adaptation.
