# Supplementary material for: Two Opposing Roles of SARS-CoV-2 RBD-Reactive Antibodies in Pre-Pandemic Plasma Samples From Elderly People in ACE2-Mediated Pseudovirus Infection
Source: Front Immunol. 2022 Jan 11;12:813240. doi: 10.3389/fimmu.2021.813240 (PMC8787138; doi:10.3389/fimmu.2021.813240)
Supplement: Supplementary file 2 [file Image_1.pdf]

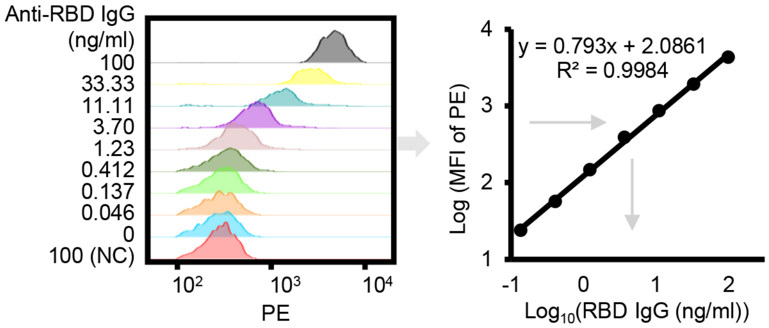

Supplementary Figure 1. Bead-based anti-RBD IgG antibody assays. Illustration of the scheme for antibody quantification using bead-based IgG antibody analysis. Standard curves of SARS-CoV-2 RBD-specific IgG were used to quantify normalized arbitrary units (AUs) of IgG levels. NC indicates protein noncoated beads used to remove signals of nonspecific binding of antibodies to beads.
