# Supplementary material for: Two Opposing Roles of SARS-CoV-2 RBD-Reactive Antibodies in Pre-Pandemic Plasma Samples From Elderly People in ACE2-Mediated Pseudovirus Infection
Source: Front Immunol. 2022 Jan 11;12:813240. doi: 10.3389/fimmu.2021.813240 (PMC8787138; doi:10.3389/fimmu.2021.813240)
Supplement: Supplementary file 3 [file Image_2.pdf]

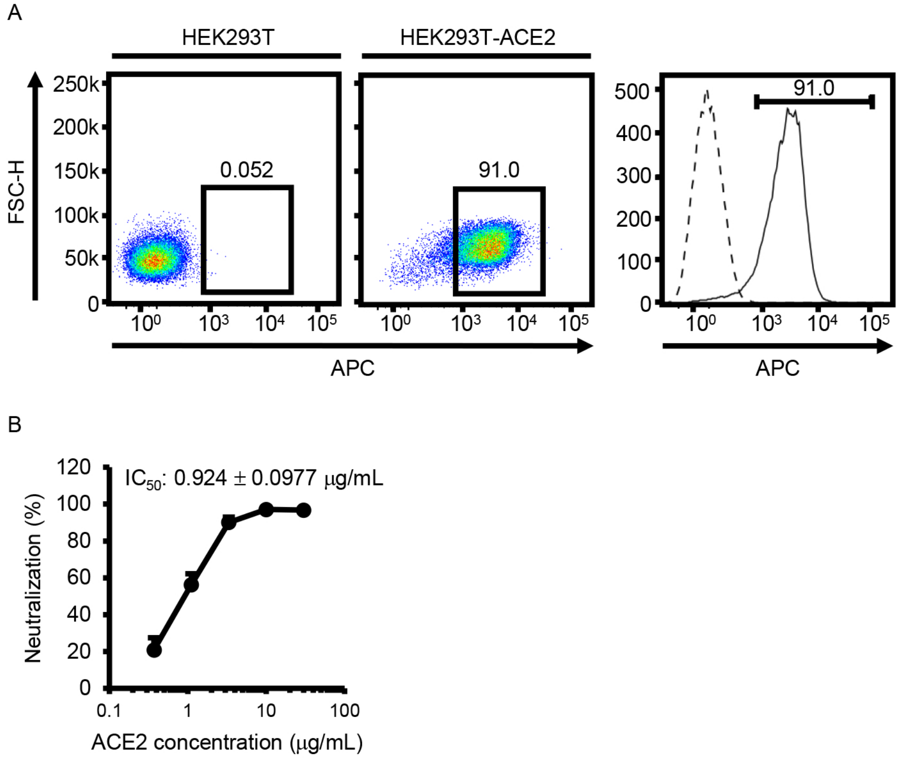

Supplementary Figure 2. ACE2-mediated neutralization of SARS-CoV-2 pseudotype virus infection. HEK293T cells were stably transfected with a plasmid encoding human ACE2 and selected by puromycin. **(A)** Flow cytometry analysis was performed using HEK293T cells and ACE2-expressing HEK293T (HEK293T-ACE2) cells stained with APC-conjugated anti-human ACE2 IgG. **(B)** The SARS-CoV-2 pseudotype neutralization assay was conducted with soluble ACE2-Fc fusion protein over a given range of concentrations (0.37~30 g/ml). The IC_{50} represents the half maximal inhibitory concentration of soluble ACE2-Fc fusion protein for neutralization.
