# Supplementary material for: Two Opposing Roles of SARS-CoV-2 RBD-Reactive Antibodies in Pre-Pandemic Plasma Samples From Elderly People in ACE2-Mediated Pseudovirus Infection
Source: Front Immunol. 2022 Jan 11;12:813240. doi: 10.3389/fimmu.2021.813240 (PMC8787138; doi:10.3389/fimmu.2021.813240)
Supplement: Supplementary file 4 [file Image_3.pdf]

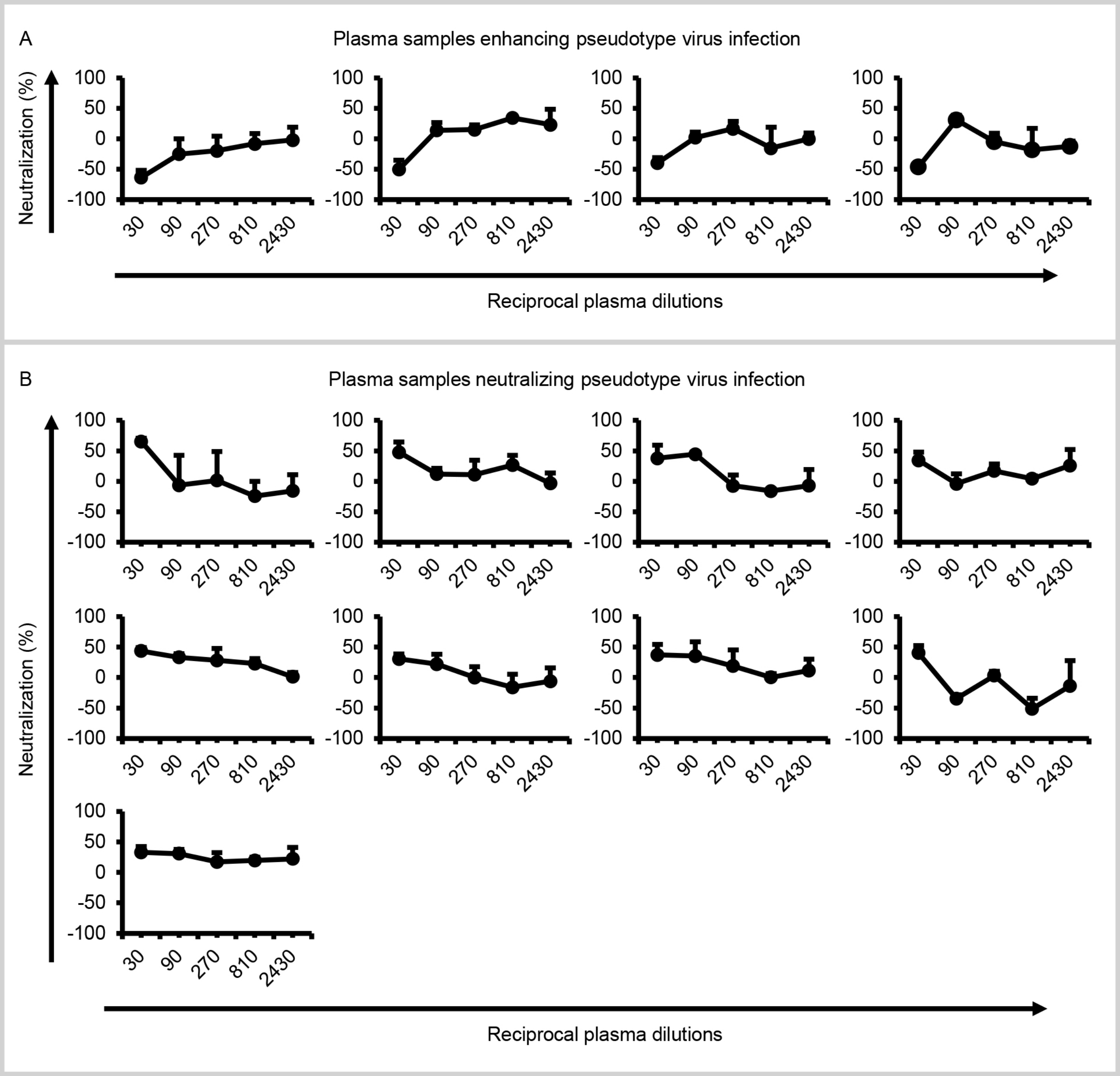

Supplementary Figure 3. SARS-CoV-2 pseudotype virus infection neutralization curves of plasma samples enhancing or neutralizing pseudotype virus infection. Raw neutralization curves for data from **Figure 4A** are shown, including for samples enhancing pseudotype virus infection (**A**, n = 4) and samples neutralizing pseudotype virus infection (**B**, n = 9). The mean and error bars are shown for each replicate.
