# Supplementary material for: Two Opposing Roles of SARS-CoV-2 RBD-Reactive Antibodies in Pre-Pandemic Plasma Samples From Elderly People in ACE2-Mediated Pseudovirus Infection
Source: Front Immunol. 2022 Jan 11;12:813240. doi: 10.3389/fimmu.2021.813240 (PMC8787138; doi:10.3389/fimmu.2021.813240)
Supplement: Supplementary file 5 [file Table_1.docx]

**Supplementary Table 1. Subject characteristics.**

| Characteristic | Number | % |
| --- | --- | --- |
| Age (years) |  |  |
| 50~59 | 3 | 2.52 |
| 60~69 | 19 | 15.97 |
| 70~79 | 82 | 68.91 |
| 80~89 | 15 | 12.61 |
| Gender |  |  |
| Men | 43 | 36.13 |
| Women | 76 | 63.87 |
